# Exploring Quality Differences in Telemedicine Between Hospital Outpatient Departments and Community Clinics: Cross-sectional Study

**DOI:** 10.2196/32373

**Published:** 2022-02-15

**Authors:** Noora Alhajri, Mecit Can Emre Simsekler, Buthaina Alfalasi, Mohamed Alhashmi, Hamda Memon, Emma Housser, Abdulhamid Mustafa Abdi, Nahed Balalaa, Maryam Al Ali, Raghda Almaashari, Shammah Al Memari, Farida Al Hosani, Yousif Al Zaabi, Shereena Almazrouei, Hamed Alhashemi

**Affiliations:** 1 College of Medicine and Health Science Khalifa University Abu Dhabi United Arab Emirates; 2 College of Engineering Khalifa University Department of Industrial and Systems Engineering Abu Dhabi United Arab Emirates; 3 Department of Family Medicine Zayed Military Hospital Abu Dhabi United Arab Emirates; 4 Department of General Surgery Sheikh Shakhbout Medical City Abu Dhabi United Arab Emirates; 5 Ambulatory Healthcare Services Abu Dhabi Health Services Company Abu Dhabi United Arab Emirates; 6 Department of Dermatology Sheikh Khalifa Medical City Abu Dhabi United Arab Emirates; 7 Abu Dhabi Public Health Center Abu Dhabi United Arab Emirates; 8 Department of Health Abu Dhabi United Arab Emirates

**Keywords:** COVID-19, patient satisfaction, technology acceptance, hospital, community clinic, video consultation, audio consultation, outpatient department, OPD, policy making, UAE

## Abstract

**Background:**

Telemedicine is a care delivery modality that has the potential to broaden the reach and flexibility of health care services. In the United Arab Emirates, telemedicine services are mainly delivered through either integrated hospital outpatient department (OPDs) or community clinics. However, it is unknown if patients’ perceptions of, and satisfaction with, telemedicine services differ between these two types of health care systems during the COVID-19 pandemic.

**Objective:**

We aimed to explore the differences in patients’ perceptions of, and satisfaction with, telemedicine between hospital OPDs and community clinics during the COVID-19 pandemic. We also aimed to identify patient- or visit-related characteristics contributing to patient satisfaction with telemedicine.

**Methods:**

In this cross-sectional study that was conducted at Abu Dhabi health care centers, we invited outpatients aged 18 years or over, who completed a telemedicine visit during the COVID-19 pandemic, to participate in our study. Patients’ perceptions of, and satisfaction with, telemedicine regarding the two system types (ie, hospital OPDs and community clinics) were assessed using an online survey that was sent as a link through the SMS system. Regression models were used to describe the association between patient- and visit-related characteristics, as well as the perception of, and satisfaction with, telemedicine services.

**Results:**

A total of 515 patients participated in this survey. Patients’ satisfaction with telemedicine services was equally high among the settings, with no statistically significant difference between the two setting types (hospital OPDs: 253/343, 73.8%; community clinics: 114/172, 66.3%; *P*=.19). Video consultation was significantly associated with increased patient satisfaction (odds ratio [OR] 2.57, 95% CI 1.04-6.33; *P*=.04) and patients’ support of the transition to telemedicine use during and after the pandemic (OR 2.88, 95% CI 1.18-7.07; *P*=.02). Patients who used video consultations were more likely to report that telemedicine improved access to health care services (OR 3.06, 95% CI 1.71-8.03; *P*=.02), reduced waiting times and travel costs (OR 4.94, 95% CI 1.15-21.19; *P*=.03), addressed patients’ needs (OR 2.63, 95% CI 1.13-6.11; *P*=.03), and eased expression of patients’ medical concerns during the COVID-19 pandemic (OR 2.19, 95% CI 0.89-5.38; *P*=.09). Surprisingly, middle-aged patients were two times more likely to be satisfied with telemedicine services (OR 2.12, 95% CI 1.09-4.14; *P*=.03), as compared to any other age group in this study.

**Conclusions:**

These findings suggest that patient satisfaction was unaffected by the health system setting in which patients received the teleconsultations, whether they were at hospitals or community clinics. Video consultation was associated with increased patient satisfaction with telemedicine services. Efforts should be focused on strategic planning for enhanced telemedicine services, video consultation in particular, for both emergent circumstances, such as the COVID-19 pandemic, and day-to-day health care delivery.

## Introduction

In the face of the COVID-19 pandemic, the world woke up to the limitations of the current health care system [1]. As an analog system, health care was scantily equipped to cope with the rapidly emerging pandemic [2]. The United Arab Emirates (UAE) health care system, like many international health care systems, had been largely based on the “in-person visit” model of care [3]. This care delivery model was challenging during the COVID-19 pandemic, given the fast spread of the virus and risk of transmission to uninfected patients who were seeking medical assessment [2,4-6]. In the UAE, it was clear that immediate action was required to transform health care delivery to a scalable digital system. [2,7]. Many hospitals and community clinics had to make a rapid transition from the previously limited scale of telemedicine to its widespread use as the primary mode of care delivery during the COVID-19 pandemic [8]. Telemedicine or telehealth, as defined by the World Health Organization, is the remote delivery of health care services and clinical information using digital technologies for the diagnosis, treatment, and prevention of disease and injuries and for the purposes of research, evaluation, and continuity of medical education [9]. During the COVID-19 pandemic, telemedicine consultations were primarily provided through (1) hospital outpatient departments (OPDs), which refer to moderately to highly integrated health care facilities providing secondary or tertiary health care services in a hospital setting, and (2) community clinics, which refer to ambulatory health care facilities that provide primary health care services. Because many of these health systems were implementing telemedicine technologies for the first time, it was unclear whether there were differences in the acceptance of this new technology for delivering health care services and whether patient satisfaction differed between the two health care systems. The acceptance of new technology was first described by Davis [10] in 1989 using the technology acceptance model (TAM). This model consists of two main constructs: (1) perceived usefulness and (2) perceived ease of use of the new technology. TAM can help us understand patients’ attitudes toward receiving clinical care through new online innovations such as telemedicine. The model also serves as a useful framework to understand intentions that influence the use of new technologies among the older generation. A more recent framework that resembled TAM was developed by Venkatesh and Davis [11] to unify technology acceptance and use. The new model, referred to as the unified theory of acceptance and use of technology, incorporated perceived usefulness into a performance expectancy construct, perceived ease of use into an effort expectancy construct, and a social influence construct, which measures the effect of social factors on acceptance and use of new technology.

Patient satisfaction has been described by the US Centers for Medicare & Medicaid Services as the patient perspective of health care services, which can be used as an objective metric to compare hospitals and other health care organizations [12]. Patient satisfaction is becoming increasingly important in all aspects of health care [13]. It is a critical metric that is frequently used to assess the efficacy of health care services [14-17]. Thus, while patient satisfaction is a proxy, it is an effective way to measure the quality of health care services as published by the US Healthcare Effectiveness Data and Information Set report [18,19]. As with traditional health care systems, survey reports of patient satisfaction can help us understand patients’ attitudes toward telemedicine [20]. Moreover, by using reports of patient satisfaction with telemedicine, we can better understand patients’ experience with health care services, promote adherence to treatment [21,22], predict health care–related behaviors [23], and predict patterns of patients’ use of new health care technologies [24]. Community clinics are considered the primary source of care for patients seeking medical care in rural and urban communities [25,26], and previous reports have indicated that community clinics struggled with the rapid shift to telemedicine during the COVID-19 pandemic, unlike well-resourced hospitals that adapted swiftly to the new mode of care delivery [27]. Additionally, previously published studies revealed differences in satisfaction among patients receiving care in different settings, such as hospitals, community clinics, and physician offices [28-31]. Thus, we sought to explore the differences in patients’ perceptions of, and satisfaction with, telemedicine between hospital OPDs and community clinics, and to propose recommendations for future telemedicine delivery through these different systems using results from this survey study. We further aimed to explore patient- or visit-related factors that contribute to increased satisfaction with telemedicine and how these factors could be applied to quality assurance in the health care system.

## Methods

### Study Design

This was a survey-based cross-sectional study conducted in December 2020 on outpatients who used telemedicine services during the COVID-19 pandemic in Abu Dhabi. Data were collected using an online survey that was sent through an internal SMS system in a manner consistent with the American Association for Public Opinion Research reporting guidelines [32]. The study followed the STROBE (Strengthening the Reporting of Observational Studies in Epidemiology) reporting guidelines [33].

### Subject Selection and Inclusion and Exclusion Criteria

The sampling method used in this study was volunteer sampling. The calculated sample size to achieve 80% power was 426 participants, with a nonresponse rate of 20%. The online survey link was sent mainly via the internal SMS system of the Abu Dhabi Department of Health (DoH) and the Abu Dhabi Health Services Company (SEHA), which are two large health regulatory authorities in Abu Dhabi with a registry of patients who visited outpatient facilities (ie, hospital OPDs and community clinics) during the COVID-19 pandemic. The inclusion criteria for participants were as follows: (1) 18 years of age or older and (2) completed a telemedicine consultation in an outpatient setting during the COVID-19 pandemic from March to December 2020. We excluded patients who had never used telemedicine services during the COVID-19 pandemic.

### Survey Development, Piloting, and Data Collection

An online survey instrument was developed using questions from the validated Telemedicine Satisfaction Questionnaire and the Telemedicine Usability Questionnaire [34,35]. The survey consisted of demographic characteristics and eight questions, rated on a 5-point Likert scale, that were revised by a team of physicians who frequently consulted patients using telemedicine services during the COVID-19 pandemic; the survey was available in English and Arabic (Multimedia Appendix 1). Two main factors were examined in this survey: patient acceptance of telemedicine and patient satisfaction with telemedicine.

The first factor, patient acceptance of telemedicine, was examined through two main constructs:

1. Perceived usefulness of telemedicine, which was evaluated through three major dimensions: improvement of access to health care, saving time and costs, and addressing health needs.
2. Perceived ease of use of telemedicine, which was assessed through the following dimension: ease of expressing clinical concerns.

The second factor, patient satisfaction with telemedicine, was assessed through four dimensions:

1. Comfort during consultation.
2. Cultural compatibility.
3. Support for the transition to telemedicine.
4. Satisfaction with telemedicine.

A pilot study was conducted using a cohort of 30 patients to assess whether the questions were comprehensible, appropriate, well-defined, and understood in a consistent manner [36]. Each patient’s information statement has also been evaluated for appropriateness and comprehension by the study investigators. The online survey instrument was built using the Microsoft Forms platform (version 2018; Microsoft Corporation). The survey was conducted over a 2-week period (ie, December 2-16, 2020); the initial invitation was sent in the first week followed by a reminder invitation in the second week to increase recruitment of subjects.

### Study Variables and Outcomes

Sociodemographic factors, including age, sex, education level, and marital status; modality of telemedicine (ie, video or audio consultation); experience with telemedicine; distance to health care facility; and type of health care system (ie, hospital OPDs or community clinics) were all self-reported by survey respondents. We compared the perceived usefulness and ease of use (ie, acceptance) of telemedicine services, as well as patient satisfaction with these services, between hospital OPDs and community clinics. We defined hospital OPDs as moderately to highly integrated health centers with high differentiation in the level of secondary and tertiary health services. We defined community clinics as ambulatory health practices that have a limited level of differentiation across services, providing mainly primary health care services. Differentiation was defined as the number of different services that the system is providing; integration has been measured by whether or not services are offered through this health system and whether or not physicians are aligned through a contractual mechanism [37,38]. These definitions were adapted from a widely recognized published taxonomy of health systems and networks by Bazzoli and colleagues [39]. Video consultation was defined as any remote clinical consultation taking place on a video platform using the camera in the patient’s smartphone, tablet, or computer, where both the patient and physician can interact in a real-time manner [40]. Audio consultation was defined as any remote clinical consultation taking place through a phone call where interaction between the physician and patient is limited to an audio conversation only [8,11,41-43]. We defined perceived usefulness as how patients perceive the usefulness of the telemedicine system regarding improvement in the performance of, and access to, health care [44]. Perceived ease of use was defined as the degree to which a person believes that using a tool or a system would be easy and free of effort [44]. Patient satisfaction was defined as a subjective measurement that reflects the difference between patient expectation and the quality of telemedicine services they have received [45,46]. We measured these constructs using a questionnaire that reflects the core theme of perceived usefulness, ease of use, and satisfaction. Lastly, we defined middle-aged as being between 40 and 59 years of age [47].

### Statistical Analysis

Differences in the perception of telemedicine services and patient satisfaction between hospital OPDs and community clinics were investigated based on various outcome variables. While the first set of outcomes was related to the perceived usefulness and ease of use of telemedicine services, the second set of outcomes assessed patients’ satisfaction with telemedicine services and whether a difference existed between these two health care systems. Descriptive statistics characterizing the survey respondents were reported as frequencies and percentages for all variables. To compare responses to survey questions among health care system types, we performed chi-square statistical tests at a significance level of .05. We used ordered logistic regression analyses to investigate the association between health care systems, modality, and outcome variables (ie, perceived usefulness and ease of use of telemedicine and patient satisfaction), adjusting for confounding factors, such as sociodemographic characteristics. A forced-entry approach was adopted to consider the variance inflation factor (VIF) diagnostic to prevent unreliable estimates of coefficients and odds ratios (ORs) due to high correlations among predictor variables. Results showed that multicollinearity was not a concern in the final models (VIF=1.1). We also checked the distribution of the responses to questions, based on a 5-point Likert scale, and found limited observations, particularly toward the extreme negative and positive ends of the scale (ie, “strongly agree” and “strongly disagree”). Considering that the number of patients who selected “strongly agree” or “strongly disagree” was not large enough to permit a meaningful statical analysis, we merged “strongly agree” and “agree” responses under a positive direction, and “strongly disagree” and “disagree” responses were merged under a negative direction. These two statements were found to involve the same attitude continuum toward the question and were collapsed into “disagreement,” “neutral,” and “agreement,” as has been done in similar previous studies [8,48,49]. Regression results were reported as ORs and 95% CIs, with *P*<.05 demonstrating statistical significance. Statistical analyses were performed using Stata Statistical Software (version 16.1; StataCorp LLC).

### Ethical Approval

We obtained ethical approval for this study from the Institutional Review Board (IRB) of Khalifa University (protocol No H21-006-2020) and the Abu Dhabi COVID-19 Research IRB Committee of the Abu Dhabi Department of Health (IRB reference number DOH/CVDC/2020/1747). A waiver for informed consent was granted due to the deidentified nature of this study.

## Results

### Overview

A total of 515 patients completed the survey, of whom 33.4% (n=172) had a telemedicine consultation through community clinics, while the majority (n=343, 66.6%) had a telemedicine consultation through hospital OPDs. The sociodemographic descriptive characteristics of the two groups were summarized and compared (Table 1).

**Table 1:** Patient sociodemographic characteristics and descriptive statistics by health care system.

Variables			Participants, n (%)^a^							*P* value
			Hospital outpatient department (n=343)		Community clinic (n=172)		Total (N=515)			
Total			343 (66.6)		172 (33.4)		515 (100)		N/A^b^	
**Sex**										
	Male	138 (40.2)		91 (52.9)		229 (44.5)		.006^c^		
	Female	205 (59.8)		81 (47.1)		286 (55.5)				
**Age range (years)**										
	≤39	133 (38.8)		82 (47.7)		215 (41.7)		.07		
	40-59	169 (49.3)		78 (45.3)		247 (48.0)				
	≥60	41 (12.0)		12 (7.0)		53 (10.3)				
**Education level**										
	High school or equivalent	118 (34.4)		63 (36.6)		181 (35.1)		.88		
	Bachelor’s degree or equivalent	172 (50.1)		83 (48.3)		255 (49.5)				
	Master’s degree, PhD, or equivalent	53 (15.5)		26 (15.1)		79 (15.3)				
**Marital status**										
	Single	54 (15.7)		34 (19.8)		88 (17.1)		.47		
	Married	260 (75.8)		126 (73.3)		386 (75.0)				
	Others (widowed or divorced)	29 (8.5)		12 (7.0)		41 (8.0)				
**Past experience with telemedicine**										
	Never used	209 (60.9)		97 (56.4)		306 (59.4)		.32		
	Used	134 (39.1)		75 (43.6)		209 (40.6)				
**Modality**										
	Audio consultation	311 (90.7)		163 (94.8)		474 (92.0)		.11		
	Video consultation	32 (9.3)		9 (5.2)		41 (8.0)				
**Employment status**										
	Employed	224 (65.3)		129 (75.0)		353 (68.5)		.03		
	Unemployed	119 (34.7)		43 (25.0)		162 (31.5)				
**Distance to health center (minutes)**										
	<30	265 (77.3)		140 (81.4)		405 (78.6)		.28		
	>30	78 (22.7)		32 (18.6)		110 (21.4)				

^a^Percentages in the “Total” row are based on the total number of participants (N=515), while percentages in all other rows are based on the total values in their respective column headings.

^b^N/A: not applicable; a statistical test was not performed on the total group.

^c^The *P* value for each group of variables is reported in the top row of each group.

### Sociodemographic Characteristics

Compared to patients who had telemedicine consultations via community clinics, patients who had telemedicine consultations via hospital OPDs were mainly female (205/343, 59.8% vs 81/172, 47.1%; *P*=.006), were middle-aged (40-59 years: 169/343, 49.3% vs 78/172, 45.3%; *P*=.07), had a college degree (bachelor’s degree: 172/343, 50.1% vs 83/172, 48.3%; *P*=.88), were married (260/343, 75.8% vs 126/172, 73.3%; *P*=.47), had no previous experience with telemedicine (209/343, 60.9% vs 97/172, 56.4%; *P*=.32), were unemployed (119/343, 34.7% vs 43/172, 25.0%; *P*=.03), and lived far from the health center (78/343, 22.7% vs 32/172, 18.6%; *P*=.28). The majority of patients who used telemedicine services in community clinics were young, male, not married, and employed and had previous experience with telemedicine. Regarding the modality of telemedicine, audio consultation was used more frequently than video consultation in both settings, and the frequency of using video consultation was higher in hospital OPDs than in community clinics; however, this result was not statistically significant (32/343, 9.3% vs 9/172, 5.2%; *P*=.11).

### Perceived Usefulness and Ease of Use of Telemedicine Services

Perceived usefulness and ease of use of telemedicine services was assessed using a multi-item approach. We assessed patients’ agreement with each statement using a 5-point Likert scale (Table 2). Perception of telemedicine usefulness and ease of use were equally high, with no statistically significant difference between the two settings.

**Table 2:** Comparison of survey responses regarding perceived usefulness and ease of use of telemedicine services provided by the health care system.

Statements and responses			Participants, n (%)^a^						*P* value	
			Hospital outpatient department (n=343)		Community clinic (n=172)		Total (N=515)			
Total			343 (66.6)		172 (33.4)		515 (100)		N/A^b^	
**Telemedicine improved access to clinical care**										
	Disagree and strongly disagree	32 (9.3)		16 (9.3)		48 (9.3)		.55^c^		
	Neutral	64 (18.7)		39 (22.7)		103 (20.0)				
	Agree and strongly agree	247 (72.0)		117 (68.0)		364 (70.7)				
**Telemedicine saved time and travel costs**										
	Disagree and strongly disagree	25 (7.3)		9 (5.2)		34 (6.6)		.29		
	Neutral	39 (11.4)		27 (15.7)		66 (12.8)				
	Agree and strongly agree	279 (81.3)		136 (79.1)		415 (80.6)				
**Telemedicine can address patients’ health care needs**										
	Disagree and strongly disagree	39 (11.4)		18 (10.5)		57 (11.1)		.61		
	Neutral	67 (19.5)		40 (23.3)		107 (20.8)				
	Agree and strongly agree	237 (69.1)		114 (66.3)		351 (68.2)				
**Medical concerns are easily expressed via telemedicine**										
	Disagree and strongly disagree	34 (9.9)		12 (7.0)		46 (8.9)		.20		
	Neutral	51 (14.8)		35 (20.3)		86 (16.7)				
	Agree and strongly agree	258 (75.2)		125 (72.7)		383 (74.4)				

^a^Percentages in the “Total” row are based on the total number of participants (N=515), while percentages in all other rows are based on the total values in their respective column headings.

^b^N/A: not applicable; a statistical test was not performed on the total group.

^c^The *P* value for each group of variables is reported in the top row of each group.

### Patient Satisfaction With Clinical Consultation

Similarly, patient satisfaction with telemedicine was assessed using a multi-item approach consisting of four satisfaction-related statements, which were rated on a 5-point Likert scale (Table 3). Patient satisfaction with telemedicine services was equally high with no statistically significant difference between the two settings.

**Table 3:** Comparison of survey responses regarding overall satisfaction with telemedicine services and clinical consultations provided by the health care system.

Statements and responses		Participants, n (%)^a^						*P* value
		Hospital outpatient department (n=343)	Community clinic (n=172)		Total (N=515)			
Total		343 (66.6)	172 (33.4)		515 (100)		N/A^b^	
**Felt comfortable consulting the physician using telemedicine services**								
	Disagree and strongly disagree	30 (8.7)		21 (12.2)		51 (9.9)		.14^c^
	Neutral	59 (17.2)		38 (22.1)		97 (18.8)		
	Agree and strongly agree	254 (74.1)		113 (65.7)		367 (71.3)		
**Telemedicine is a culturally appropriate way to receive health care services**								
	Disagree and strongly disagree	33 (9.6)		19 (11.0)		52 (10.1)		.88
	Neutral	81 (23.6)		40 (23.3)		121 (23.5)		
	Agree and strongly agree	229 (66.8)		113 (65.7)		342 (66.4)		
**Support the transition to telemedicine services during and after the pandemic**								
	Disagree and strongly disagree	33 (9.6)		15 (8.7)		48 (9.3)		.44
	Neutral	67 (19.5)		42 (24.4)		109 (21.2)		
	Agree and strongly agree	243 (70.8)		115 (66.9)		386 (69.5)		
**Satisfied with the quality of telemedicine services**								
	Disagree and strongly disagree	31 (9.0)		18 (10.5)		49 (9.5)		.19
	Neutral	59 (17.2)		40 (23.3)		99 (19.2)		
	Agree and strongly agree	253 (73.8)		114 (66.3)		367 (71.3)		

^a^Percentages in the “Total” row are based on the total number of participants (N=515), while percentages in all other rows are based on the total values in their respective column headings.

^b^N/A: not applicable; a statistical test was not performed on the total group.

^c^The *P* value for each group of variables is reported in the top row of each group.

### Multivariate Analysis

In the multivariate model, the use of video consultation was significantly associated with increased perceived usefulness and ease of use of telemedicine. As compared to patients who had audio calls, patients who had video consultations were 3 times more likely to report that telemedicine improved access to health care services (OR 3.06, 95% CI 1.17-8.03; *P*=.02), 5 times more likely to report that telemedicine reduced waiting times and travel costs (OR 4.94, 95% CI 1.15-21.19; *P*=.03), and 2.63 times more likely to report that telemedicine can address patients’ medical needs (OR 2.63, 95% CI 1.13-6.11; *P*=.03). There was no statistically significant association between the type of health care system (ie, hospital OPD vs community clinic) and patients’ perceptions toward telemedicine (Table 4). Surprisingly, middle-aged patients were more likely to have higher perceived usefulness of telemedicine, indicating greater acceptance of the new technology-based model of care delivery.

**Table 4:** Adjusted multivariate analysis for perceived usefulness and ease of use of telemedicine.

Variables			Telemedicine improved access to clinical care				Telemedicine saved time and travel costs				Telemedicine addressed patients’ health care needs				Telemedicine eased expression of medical concerns		
			OR^a^ (95% CI)		*P* value		OR (95% CI)		*P* value		OR (95% CI)		*P* value		OR (95% CI)		*P* value
Health care system (hospital vs clinic)			1.20 (0.80-1.81)		.37		1.01 (0.63-1.64)		.94		1.05 (0.71-1.56)		.80		1.03 (0.67-1.56)		.91
Modality (video call vs audio call)			3.06 (1.17-8.03)		.02		4.94 (1.15-21.19)		.03		2.63 (1.13-6.11)		.03		2.19 (0.89-5.38)		.09
Sex (female vs male)			0.88 (0.58-1.33)		.54		1.31 (0.81-2.12)		.27		1.21 (0.80-1.82)		.36		1.06 (0.69-1.63)		.79
**Age range (years)**																	
	≤39 vs ≥60	1.48 (0.78-2.84)		.23		1.82 (0.87-3.82)		.11		1.32 (0.68-2.56)		.42		0.88 (0.42-1.83)		.73	
	40-59 vs ≥60	1.95 (1.02-3.71)		.04		3.03 (1.12-6.46)		.004		1.30 (0.68-2.50)		.43		1.14 (0.55-2.37)		.73	
**Education level**																	
	College degree vs high school diploma	0.78 (0.50-1.21)		.27		0.79 (0.48-1.32)		.37		0.75 (0.49-1.16)		.20		0.76 (0.48-1.20)		.24	
	PhD degree vs high school diploma	0.99 (0.52-1.90)		.99		2.06 (0.88-4.84)		.10		1.07 (0.58-1.98)		.82		0.96 (0.50-1.85)		.90	
**Marital status**																	
	Married vs single	0.87 (0.50-1.50)		.62		1.21 (0.66-2.20)		.54		1.07 (0.63-1.80)		.80		0.95 (0.55-1.66)		.87	
	Others vs single	1.08 (0.46-2.56)		.85		1.22 (0.46-3.28)		.69		1.09 (0.47-2.56)		.83		1.09 (0.43-2.75)		.85	
Past experience with telemedicine (used vs never used)			0.93 (0.63-1.38)		.72		0.99 (0.63-1.59)		>.99		1.17 (0.79-1.71)		.44		0.77 (0.52-1.16)		.22
Employment status (employed vs unemployed)			1.22 (0.76-1.94)		.41		0.75 (0.43-1.33)		.33		0.93 (0.59-1.49)		.78		0.88 (0.53-1.45)		.67
Distance to health center (>30 min vs <30 min)			1.05 (0.65-1.68)		.85		0.89 (0.52-1.52)		.66		0.95 (0.60-1.49)		.82		0.85 (0.53-1.36)		.49

^a^OR: odds ratio.

Additionally, when compared to patients who had audio consultations, patients who had video consultations were 2.88 times more likely to support the transition to telemedicine services during and after the pandemic (OR 2.88, 95% CI 1.18-7.07; *P*=.02) and 2.57 times more satisfied with telemedicine services (OR 2.57, 95% CI 1.04-6.33; *P*=.04). Similarly, when compared to patients aged 60 years or older, middle-aged patients were 2 times more likely to be satisfied with telemedicine services (OR 2.12, 95% CI 1.09-4.14; *P*=.03). Additionally, when compared to employed patients, unemployed patients were more likely to be satisfied with telemedicine (OR 0.57, 95% CI 0.35-0.94; *P*=.03). However, sex, education level, marital status, experience with telemedicine, and distance to health care center were not significantly associated with patient satisfaction with telemedicine services during the pandemic (Table 5).

**Table 5:** Adjusted multivariate analysis for patient satisfaction with clinical consultations.

Variables			Felt comfortable consulting the physician using telemedicine services					Telemedicine is a culturally appropriate way to receive health care services					Support the transition to telemedicine services during and after the pandemic					Satisfied with the quality of telemedicine services		
			OR^a^ (95% CI)		*P* value		OR (95% CI)			*P* value		OR (95% CI)			*P* value		OR (95% CI)			*P* value
Health care system (hospital vs clinic)			1.44 (0.96-2.16)		.08		1.06 (0.72-1.57)			.76		1.11 (0.75-1.65)			.61		1.32 (0.88-1.98)			.18
Modality (video call vs audio call)			2.25 (0.96-5.26)		.06		1.58 (0.75-3.32)			.23		2.88 (1.18-7.07)			.02		2.57 (1.04-6.33)			.04
Sex (female vs male)			1.06 (0.70-1.60)		.79		1.11 (0.74-1.65)			.61		1.02 (0.68-1.53)			.93		1.06 (0.70-1.61)			.78
**Age range (years)**																				
	≤39 vs ≥60	1.57 (0.80-3.11)		.19		1.11 (0.58-2.13)			.74		1.63 (0.84-3.16)			.15		1.46 (0.75-2.85)			.27	
	40-59 vs ≥60	1.72 (0.88-3.36)		.11		1.20 (0.63-2.26)			.58		1.80 (0.94-3.46)			.08		2.12 (1.09-4.14)			.03	
**Education level**																				
	College degree vs high school diploma	0.76 (0.48-1.20)		.24		0.71 (0.47-1.09)			.12		0.76 (0.49-1.19)			.23		0.67 (0.43-1.06)			.09	
	PhD degree vs high school diploma	0.89 (0.47-1.66)		.71		1.01 (0.54-1.88)			.98		0.96 (0.51-1.78)			.89		0.96 (0.50-1.83)			.90	
**Marital status**																				
	Married vs single	0.90 (0.53-1.54)		.70		0.89 (0.53-1.50)			.66		1.03 (0.61-1.74)			.90		0.88 (0.51-1.52)			.65	
	Others vs single	1.32 (0.51-3.41)		.56		0.59 (0.26-1.30)			.19		1.25 (0.53-2.96)			.62		0.91 (0.37-2.25)			.84	
Past experience with telemedicine (used vs never used)			1.01 (0.68-1.50)		.96		1.04 (0.72-1.52)			.83		1.08 (0.73-1.59)			.72		1.05 (0.70-1.56)			.83
Employment status (employed vs unemployed)			0.73 (0.45-1.18)		.20		0.92 (0.58-1.44)			.70		0.77 (0.48-1.24)			.29		0.57 (0.35-0.94)			.03
Distance to health center (>30 min vs <30 min)			0.79 (0.50-1.24)		.31		0.90 (0.58-1.40)			.63		1.06 (0.66-1.71)			.80		0.81 (0.51-1.30)			.38

^a^OR: odds ratio.

## Discussion

### Principal Findings

The front lines of medicine in many health care systems, including primary care clinics, were severely disrupted during the COVID-19 emergency. Despite the initial shock, many health systems were quick to adapt to the use of digital technologies; however, for some health systems, the transition has been smoother and faster than for others. During this crisis, telemedicine services have proven to be an integral part of the global public health response and showed capacity to act as a “safety net” for patients when properly reinforced [50-52]. In this paper, we have critically examined patients’ acceptance of telemedicine as a new technology for health care delivery and patient satisfaction with telemedicine services across two common health system types: hospitals and community clinics. We have further explored patient characteristics and factors that predict satisfaction with telemedicine services. The Institute of Medicine recommends assessing the quality of health systems’ services either through patient satisfaction reports or through technical and professional assessment [52]; therefore, we used patient satisfaction survey results as a proxy to evaluate telemedicine quality across two types of health systems. Results from this study highlight three key findings: (1) there were no statistically significant differences in patient satisfaction between hospitals and community clinics regarding telemedicine services, (2) video consultation was significantly associated with increased patient satisfaction with telemedicine during the pandemic, and (3) being middle-aged was a significant predictor for patient satisfaction with telemedicine services, indicating higher acceptance of digital health among this age group.

Our first key finding suggests that perception of usefulness of telemedicine services, ease of use of these services, and satisfaction with these services were equally high among patients who had their telemedicine consultations in either hospitals or community clinics; this indicated similar quality of digital health services across these two types of health systems. Digital health innovations in community clinics have existed for some time, although the extent to which they are used vary greatly between countries. It is time to embrace these new technologies and increase the use of these innovations for patient management and follow-up, especially in community clinics, and to not fundamentally limit their use in integrated hospitals. Our results showed that 66.6% of all telemedicine consultations occurred mainly through hospitals, while only 33.3% occurred through community clinics. These findings highlight the need to increase the implementation and delivery of digital health innovations, particularly in health facilities, which are often considered the first point of contact for patients seeking medical care [53]. There is a need to build on the current status quo and accelerate the rollout of these digital technologies for routine use in primary health care settings, such as community clinics, to increase access to health care services.

Community clinics are a pivotal part of the public health system that could significantly improve access to health care services for the most vulnerable segments of the population if properly implemented within communities [26]. The pandemic has served as a catalyst to propel the use of telemedicine technologies into routine practice, and there is a significant amount of optimism surrounding this step. Results from recent studies in telemedicine showed that many patients with long-term chronic conditions prefer remote monitoring; thus, it is vital to opt for digital transformation of primary care services and follow-up care [53,54].

The second key finding from this paper is that video consultation, as compared to audio consultation, was significantly associated with improved perceived usefulness of telemedicine and higher levels of patient satisfaction. It comes as no surprise that patients favored video consultation over audio consultation, as it breaks the psychological barrier, eases guided remote physical examination of the patient, facilitates clinical decision-making, and eases expression of patients’ concerns [55]. Moreover, the new generation of “digital native” patients are experienced in digital technologies and are comfortable communicating via virtual platforms, such as Skype, FaceTime, and Zoom [56]. Therefore, it is intuitive to introduce a telemedicine curriculum in medical schools and propose a model of education to effectively leverage telemedicine technologies and artificial intelligence in patient management [57,58]. Mainstreaming telemedicine and video applications in health systems could reduce health care disparities [59]. The surge in developing telemedicine applications with video call features is one of the most defining trends in this decade and will have a profound impact on socioeconomic and geopolitical realities, in particular in low- and middle-income countries (LMIC) [60,61]. With these telemedicine platforms, it is now possible to widen telemedicine use to remote geographical areas in LMIC and war zones. Policies advocating for the use of video consultation for certain patient categories should be implemented at the grassroots level. Such policies can specify, for instance, regulations for acute and chronic patient management; they can also specify recommendations for new or follow-up patient care and whether it is recommended to have an in-person visit, video consultation, or audio consultation, depending on the initial evaluation using the Triage and Acuity Scale [62].

The third key finding from this paper is that middle-aged patients had a higher perceived usefulness and satisfaction with telemedicine when compared to patients in other age groups. In Abu Dhabi, at least one in five middle-aged patients showed acceptance of telemedicine use, possibly because telemedicine is convenient, safe, efficient, and cost-effective and can improve work-life balance [63,64]. However, we expected to see a satisfaction gap where Millennials—also known as Generation Y, born between 1981 and 1996—have higher levels of satisfaction, as they are labeled the “technology-savvy generation,” relying heavily on technology and social media platforms for communication and addressing their life needs [65-67]. Our findings showed that patients belonging to Generation X—also known as the Baby Bust generation, born between 1960 and 1980—were the most satisfied with telemedicine. Thus, we propose that when studying patient experience with telemedicine, generational differences should be investigated further.

### Strengths and Limitations

This study has several strengths. To our knowledge, this is the first study to explore the differences in patient acceptance of, and satisfaction with, telemedicine between different health system types using patient survey results. Second, the study used a piloted and validated questionnaire that was derived from previously published studies in peer-reviewed journals. Third, the study measured the effect of telemedicine modalities (ie, video or audio consultation) on patient acceptance of, and satisfaction with, telemedicine, which is informative for decision-making policies.

Despite these strengths, the study has several limitations. First, this was a cross-sectional study capturing data entries at a single point in time with no comparison between in-person visits before and during the COVID-19 pandemic. Yet, while we felt that it was not possible to have an equal comparison since the number of in-person visits were very scarce during the pandemic due to the challenging situation, we plan to investigate this in future studies. Second, patient preference for telemedicine versus in-person visits was not captured, in addition to preference for video versus audio consultations. Third, the study did not investigate the factors influencing the age gap in telemedicine satisfaction and acceptance; however, we plan to address this as well in future studies. Moreover, this study did not measure the behavioral intention toward using video versus audio consultations. We aim to explore these factors in future studies. Lastly, our results could have been subjected to self-selection bias, as it is possible that patients who favored telemedicine or those who were more tech savvy were the ones who were motivated to participate in the study.

### Conclusions

During the COVID-19 pandemic, telemedicine played a positive role in improving health system and patient outcomes. While there are several studies in the literature that evaluated patient experience with telemedicine, there are no studies that evaluated the difference in patient satisfaction with telemedicine between different system types. Our study findings showed that patient satisfaction with telemedicine did not vary between different health care settings; however, further studies are needed to objectively assess the differences in quality of telemedicine between these two systems. This study also demonstrated that video consultation was associated with higher patient satisfaction and improved teleconsultation experience. This finding may support and accelerate the rollout of video applications for all health care systems. Moving forward, it is vital to augment digital health innovations in community clinics in order to create a sustainable and effective health care system that is capable of coping with generational and technological trends.

## Appendix

Multimedia Appendix 1 Patient survey.

## Abbreviations

DoH: Department of Health
IRB: Institutional Review Board
LMIC: low- and middle-income countries
OPD: outpatient department
OR: odds ratio
SEHA: Abu Dhabi Health Services Company
STROBE: Strengthening the Reporting of Observational Studies in Epidemiology
TAM: technology acceptance model
UAE: United Arab Emirates
VIF: variance inflation factor
