# Supplementary material for: Exploring Quality Differences in Telemedicine Between Hospital Outpatient Departments and Community Clinics: Cross-sectional Study
Source: JMIR Med Inform. 2022 Feb 15;10(2):e32373. doi: 10.2196/32373 (PMC8849258; doi:10.2196/32373)
Supplement: Multimedia Appendix 1 [file medinform_v10i2e32373_app1.docx]

**Patient's questionnaire**

This survey has been designed to explore patients’ perception and satisfaction of telemedicine technology compared to traditional face-to-face medical visits. This study does not pose any physical or psychological risk. We will not be collecting any information that can identify you. Participation in this study is completely voluntary. If you decide not to participate, there will not be any negative consequences. If you wish to exit after you started the survey, simply close the survey.

Thank you for your time,

By clicking START you agree to participate and acknowledge that you understand the content of this study.

START

EXIT

1.Gender

1. Male
2. Female

2.Age

1. ≥39
2. 40-59
3. 60+

3.Education level

1. High School or below
2. Bachelor’s degree
3. Masters/PhD

4.Martial Status

1. Married
2. Single
3. Others (Divorced, widowed)

5.Current employment

1. Unemployed
2. Employed

6. On average, how long does it take you to reach your preferred healthcare facility, when having in-person medical visits?

1. >30 mins
2. 30-60 mins

**The following questions are based on your last telemedicine consultation:**

7. How did you have your telemedicine consultation?

1. Audio Call
2. Video call

8. Where did you receive your LAST telemedicine consultation?

1. Hospital OPD
2. Community Clinic

9.Telemedicine improved my access to healthcare services

1. Strongly disagree
2. Disagree
3. Neutral
4. Agree
5. Strongly Agree

10.Telemedicine saved me time and costs traveling to a hospital or clinic.

1. Strongly disagree
2. Disagree
3. Neutral
4. Agree
5. Strongly Agree

11.Telemedicine can address my healthcare needs

1. Strongly disagree
2. Disagree
3. Neutral
4. Agree
5. Strongly Agree
6. Strongly Agree

12. I felt It was easy to express my medical concerns through telemedicine

1. Strongly disagree
2. Disagree
3. Neutral
4. Agree
5. Strongly Agree

13. I feel comfortable communicating with the doctor using the telemedicine system

1. Strongly disagree
2. Disagree
3. Neutral
4. Agree
5. Strongly Agree

14. I fully support the transition to telemedicine service during and after the pandemic.

1. Strongly disagree
2. Disagree
3. Neutral
4. Agree
5. Strongly Agree

15.Telemedicine is a culturally acceptable way to receive healthcare services

1. Strongly disagree
2. Disagree
3. Neutral
4. Agree
5. Strongly Agree

16.Overall, I am satisfied with this telemedicine system

1. Strongly disagree
2. Disagree
3. Neutral
4. Agree
5. Strongly Agree
